# Supplementary material for: Estimated glomerular filtration rate equations in people of self-reported black ethnicity in the United Kingdom: Inappropriate adjustment for ethnicity may lead to reduced access to care
Source: PLoS One. 2021 Aug 12;16(8):e0255869. doi: 10.1371/journal.pone.0255869 (PMC8360513; doi:10.1371/journal.pone.0255869)
Supplement: Attachment — Submitted filename: PLosOnereviewUKpaper.docx [file pone.0255869.s001.docx]

**PLOS-ONE MANUSCRIT**

**Dear Editor,**

I read with interest the paper entitled ‘’ **Estimated glomerular filtration rate equations in Blacks British people: inappropriate adjustement for ethnicity may led to reduced access to care’’**

Ronvick Gama et al studies 2333 participants (314 black and 2019 White) and found that eGFR equations using ethnicity correction in Black British people overestimated mGFR. Although interesting, I have some several concerns.

1. Participants were recruited from hospital database; did they have acute kidney disease or CKD?
2. Authors must clarify which statistical analysis was used to evaluate the performance of equations versus mGFR? I suggest author to perform the Bland and Altman analysis in order to illustrate performance of the different equations in figure.
3. Authors do not explain if mGFR and eGFR were performed at the same time? Additional explanations are necessary for a better understanding of the paper.
4. Also what is the operational definition of Blacks British people? How long they stay in UK? Black people living in UK are they comparable to those living in Africa? After how long time can you expected to see changes in muscle mass?
5. Are the both equations comparable, especially in healthy subjects?

Finally, I think this paper needs major revisions before being published.

Reviewer
