# Supplementary material for: Estimated glomerular filtration rate equations in people of self-reported black ethnicity in the United Kingdom: Inappropriate adjustment for ethnicity may lead to reduced access to care
Source: PLoS One. 2021 Aug 12;16(8):e0255869. doi: 10.1371/journal.pone.0255869 (PMC8360513; doi:10.1371/journal.pone.0255869)
Supplement: Attachment — Submitted filename: Ethnicity GFR Study - PLOS1 reviewers comments 7_7_21.docx [file pone.0255869.s003.docx]

The article has been largely improved. The authors should be congratulated for that.

Thank you for the kind comments.

I have three minor comments
1) The authors wrote: "This is the largest study exploring the accuracy and impact of eGFR equations in people of African and Afro-Caribbean ancestry outside of Africa or the USA". This is incorrect. Flamant et al (ref 11) studied more European black subjects (n=302) than in the current analysis (n=266). Please tone down.

We apologise for this error, and have modified to the following. ‘This is one of the largest studies’

2) I agree the authors keep their coefficient, even if I also agree with Reviewer that correction should be at the creatinine level, not the GFR level. However, I question the clinical relevance of the correction 1.018. Is it really significant from a clinical perspective? To be discussed.

We agree with this comment and have added that this is unlikely to have clinical importance Page 17 Line 13.

3) It must be reminded that the EKFC equation has the advantage to estimate GFR with the same equation in adults and children (even if no children were included in the current analysis). It seems to me that the EKFC equation performs quite good in most of analyses. It could be a bit more discussed.

Thank you for this suggestion. We have added more detail to the discussion – Page 22 Line 20.


Reviewer #1: Thank you for addressing my comments. I would suggest three other minor revisions:
1) in Table 2, serum creatinine should be reported separately for males and females, as the differences between genders are large

This detail has now been added to Table 2.

2) As the use of a "race coefficient" is highly debated in the US, I would not recommend to calculate yet another "race coefficient" of 1.018 for Black British subjects. As I have said before, the adjustment should be at the creatinine level, not at the GFR-level, which falsely suggests that there are differences in GFR between Black and White people.

We completely concur with this comment and have amended the discussion to suggest that it should not be used in clinical practice.

3) the authors report "mean" bias in the tables, but this can be largely influenced by outliers (which are always present). In many other studies, "median" bias was the more commonly reported statistic. I would suggest to replace mean bias by median bias (or at least, add median bias).

We have added median bias to the tables.

Reviewer #2: All comments have been well addressed. But, some minors modifications are needed.

For example, line 8, page 5 the design of the study is observational cross sectional study (not a cohort study).

Line 24, page 18 to refer to the study of Bukabau et al, authors should add in the sentence ''East, Central, West and South Africa report'' that is because the Democratic Republic of the Congo is in Central Africa not in East Africa.

Thank you for highlighting these inaccuracies which we have now amended.

Reviewer #4: The authors have mostly responded to reviewer concerns. One minor point remains:

1. In the first paragraph of the results, please explicitly list "predefined confounders".

Thank you for this suggestion. We have now added the predefined confounders again in this section.
